# Reduced cerebral blood flow and white matter hyperintensities predict poor sleep in heart failure

**DOI:** 10.1186/1744-9081-9-42

**Published:** 2013-10-30

**Authors:** Michael L Alosco, Adam M Brickman, Mary Beth Spitznagel, Erica Y Griffith, Atul Narkhede, Ronald Cohen, Lawrence H Sweet, Joel Hughes, Jim Rosneck, John Gunstad

**Affiliations:** 1 Department of Psychology, Kent State University, Kent, OH, USA; 2 Department of Neurology, Taub Institute for Research on Alzheimer’s Disease and the Aging Brain, College of Physicians and Surgeons, Columbia University, New York, NY, USA; 3 Department of Psychiatry, Summa Health System Akron City Hospital, Akron, OH, USA; 4 Departments of Neurology Psychiatry and the Institute on Aging, Center for Cognitive Aging and Memory, University of Florida, Florida, Gainesville, USA; 5 Department of Psychology, University of Georgia, Athens, USA

**Keywords:** Sleep quality, Heart failure, MRI, Brain perfusion, White matter hyperintensity

## Abstract

**Background:**

Poor sleep is common in heart failure (HF), though mechanisms of sleep difficulties are not well understood. Adverse brain changes among regions important for sleep have been demonstrated in patients with HF. Cerebral hypoperfusion, a correlate of sleep quality, is also prevalent in HF and a likely contributor to white matter hyperintensities (WMH). However, no study to date has examined the effects of cerebral blood flow, WMH, and brain volume on sleep quality in HF.

**Methods:**

Fifty-three HF patients completed the Pittsburgh Sleep Quality Index and underwent brain magnetic resonance imaging to quantify brain and WMH volume. Transcranial Doppler ultrasonography assessed cerebral blood flow velocity of the middle cerebral artery (CBF-V of the MCA).

**Results:**

75.5% of HF patients reported impaired sleep. Regression analyses adjusting for medical and demographic factors showed decreased CBF-V of the MCA and greater WMH volume were associated with poor sleep quality. No such pattern emerged on total brain or regional volume indices.

**Conclusions:**

Decreased cerebral perfusion and greater WMH may contribute to sleep difficulties in HF. Future studies are needed to confirm these findings and clarify the effects of cerebral blood flow and WMH on sleep in healthy and patient samples.

## Background

Heart failure (HF) affects greater than 5 million American adults and is associated with poor outcomes such as cognitive impairment, decreased quality of life, rehospitalizations, and increased morbidity and mortality risk [1-3]. As many as 80% of HF patients also report experiencing sleep difficulties [4] that are multifaceted in nature, including difficulties falling asleep, insomnia, interrupted sleep at night (e.g., nocturia), restless sleep, and breathing difficulty during sleep [5-10]. Poor sleep quality in HF, in-turn, is associated with decreased quality of life, depression, impaired self-care, and decreased participation in key treatment recommendations (e.g., physical activity) [11,12].

Much attention has been paid to correlates and modifiers of poor sleep quality in HF. Sleep quality in HF is affected by an array of demographic, medical, and clinical factors. Demographic variables that negatively influence sleep in HF include older age and being female [5,7,13]. In terms of medical factors, taking beta-blockers and medical comorbidities such as respiratory disease, stroke, hypertension, and coronary artery disease have been linked with sleep disturbances [5]. Many of these comorbidities are theorized to negatively affect circadian rhythms subsequent to associated disabilities and limited daytime activity [14]. Known psychosocial modifiers of sleep in HF include depression, anxiety, and perceived health status [5,13]. Despite these findings, the literature on factors that influence sleep quality in HF is not entirely consistent [5,7,13], suggesting poor sleep quality in HF may be more complicated than typically believed.

Although not yet examined, a likely mechanism of poor sleep in HF may involve structural and functional brain abnormalities. Adverse brain changes such as brain atrophy, white matter hyperintensities (WMH), and cerebral hypoperfusion are common in HF and believed to underlie cognitive impairment in this population [15,16]. Interestingly, recent work also shows poor sleep is associated with reduced cognitive function in HF [11] and HF patients exhibit reduced structural (e.g., atrophy) and functional (e.g., impaired axonal projections) brain integrity among key brain regions that help regulate sleep (e.g., raphe magnus, hypothalamus, medial temporal lobe structures) [17-20]. Cerebral hypoperfusion is commonly proposed to underlie adverse brain changes in HF, including white matter hypertintensities, and may also impact sleep quality in HF through its impact on the brain and sleep regulation [15,21]. Indeed, altered cerebral hemodynamics, brain atrophy, and WMH have all been independently correlated with sleep difficulties and regulation (e.g., excessive daytime sleepiness, difficulties with breathing during sleep, arousals during sleep) in other populations [19,22-25].

Despite these findings, the mechanisms of sleep difficulties in HF patients remain poorly understood. The purpose of the current study was to examine the effects of cerebral blood flow, WMH, and total brain volume on sleep disturbances in older adults with HF. This study also examined the possible association between sleep quality and volumes of regional brain structures believed to be important for sleep (e.g., thalamus and brain stem) [19,26]. Based on past work in other samples, we hypothesized that adverse structural brain changes (e.g., greater WMH, smaller total and regional brain volume) would be associated with greater sleep disturbances in HF due to their known role in sleep regulation. We also hypothesized that reduced cerebral blood flow would predict poorer sleep quality in HF in light of its known association with brain insult (e.g., WMH) in this population.

## Methods

### Participants

A total of 77 patients with HF were recruited for a NIH funded study examined cardiac rehabilitation and neurocognitive outcomes in HF. All participants were recruited from primary care and cardiology practices at Summa Health System in Akron, Ohio, and reflect the HF population receiving treatment at that facility. Data collection for this study occurred between 2009 and 2012. Throughout the study, HF participants completed assessments over a one-year period. For this cross-sectional study, only baseline assessments among a subset of non-cardiac rehabilitation controls that underwent neuroimaging were examined.

The participant sample size of the current study was reduced to 53 due to missing data on study variables and on individual items of the Pittsburgh Sleep Quality Index (PSQI) used to calculate the global composite, and/or invalid responses on the PSQI. Invalid responses included >100% in the calculation of component number 4 of the PSQI (i.e., percent of time spent sleeping while in bed). Excluded participants did not differ in age (*t*(75) = -1.50, *p* = 0.14), gender (χ^2^ (df =1) = 1.02, *p* = 0.31), education (*t*(75) = -1.19, *p* = 0.24), cerebral blood flow velocity (*t*(68) = 0.01, *p* = 0.99) or in terms of medical comorbid history, including hypertension (χ^2^ (df = 1) = 3.84, *p* = 0.05), sleep apnea (χ^2^ (df = 1) = 0.06, *p* = 0.80), and diabetes (χ^2^ (df = 1) = 2.32, *p* = 0.13). However, excluded participants had a higher left ventricular ejection fraction (*t*(74) = 2.45), *p* = 0.02; M (SD) = 49.43 (14.34) vs. 40.98 (13.57)).

Strict inclusion/exclusion criteria were chosen for entry into the larger NIH funded study. Inclusion criteria were as follows: ages of 50–85 years, English as a primary language, and a diagnosis of New York Heart Association (NYHA) class II or III at the time of enrollment. Potential participants were excluded for any contraindications to magnetic resonance imaging (MRI) (e.g., pacemaker), history of head injury with more than 10 minutes loss of consciousness, Axis I psychiatric disorders (e.g. schizophrenia, bipolar disorder), substance abuse and/or dependence, and renal failure. Participants were also excluded for a history of significant neurological disorder (e.g., dementia) and the current sample exhibited an average Mini Mental Status Examination score of 27.72 (SD = 1.96). Inclusion and exclusion criteria were determined through self-report and corroborated and supplemented by a thorough medical record review. See Table 1 for demographic and medical information.

**Table 1:** Demographic, medical, and clinical characteristics of 53 older adults with heart failure

**Demographic characteristics**	
Age, mean (SD)	69.81 (8.79)
Sex (% Women)	37.7
Race (% Caucasian) (*N* = 51)	86.8
Education, mean years (SD)	14.17 (2.87)
**Medical and clinical characteristics**	
LVEF, mean (SD)	40.98 (13.57)
Sleep Medication (%)	7.5
Diabetes (%)	24.5
Hypertension (%)	66.0
Sleep Apnea (%)	26.4
Depression (%)	17.0
2-minute step test, mean (SD)	66.02 (22.56)
CBF-V MCA, cm/s mean (SD)	42.89 (12.94)
Global PSQI, mean (SD)	8.42 (3.57)

*Note*. *LVEF* left ventricular ejection fraction, *CBF*-*V MCA* cerebral blood flow velocity of the middle cerebral artery, *PSQI* Pittsburgh sleep quality index.

### Measures

#### Sleep quality

The Pittsburgh Sleep Quality Index (PSQI) was used to assess sleep quality in the current sample [27]. The PSQI is a 19-item self-report measure that generates 7 components of sleep quality, including subjective sleep quality, sleep latency, sleep duration, habitual sleep efficiency, sleep disturbances, use of sleeping medication, and daytime dysfunction. The sum of these components yields a global PSQI score that range from 0 to 21. Higher scores reflect poorer sleep quality and a score >5 is a sensitive and specific predictor of impaired sleep [27]; this cut score was used to help characterize the sample and the continuous global PSQI served as the dependent variable. The PSQI demonstrates strong psychometric properties, including internal consistency, test-retest reliability, and is widely used in medical populations including HF [5,11].

#### Neuroimaging

Whole-brain, high-resolution 3D T1-weighted images (Magnetization Prepared Rapid Gradient-Echo, MPRAGE) were acquired on a Siemens Symphony 1.5 Tesla magnetic resonance imaging scanner for morphologic analysis. Twenty-six slices were acquired in the sagittal plane with a 230×100 mm field of view. The acquisition parameters were as follows: Echo time (TE) = 17, repetition time (TR) = 360, acquisition matrix = 256×100, and slice thickness = 5 mm. Whole-brain FLAIR images were also acquired to quantify WMH. For the FLAIR images, twenty-one 5-mm slices were acquired with TR = 8500, TI = 2500, Flip Angle = 150 degrees, TE = 115, and FOV = 220×75.

Morphometric analysis of brain structure was completed with FreeSurfer Version 5.1 (http://surfer.nmr.mgh.harvard.edu). Detailed methodology for regional and total volume derivation has been described in detail previously [28-30]. FreeSurfer was used to perform image preprocessing (e.g. intensity normalization, skull stripping), then to provide both cortical and subcortical volume measures using the surface stream and the subcortical segmentation stream respectively. FreeSurfer performs such parcellations by registering images to a probabilistic brain atlas, built from a manually labeled training set, and then using this probabilistic atlas to assign a neuroanatomical label to each voxel in an MRI volume. Total brain volume, total gray matter volume, and volume of the thalamus and brain stem were all automatically derived with the subcortical processing stream (i.e., “aseg.stats”). Intracranial volume was also automatically derived and served as a covariate in analyses examining MRI indices in order to control for interindividual differences in head size. A summary composite was computed for the left and right hemisphere volumes of the thalamus.

Total WMH volume was derived by a three-step operator-driven protocol that has been described in detail previously [31]. Briefly, in Step 1, a threshold was applied to each FLAIR image to label all voxels that fell within the intensity distribution of hyperintense signal. In Step 2, gross regions-of-interest (ROI) were drawn manually to include WMH but to exclude other regions (e.g., dermal fat) that have similar intensity values. In Step 3, a new image is generated that contains the intersection of voxels labeled in Step 1 and those labeled in Step 2. The resulting image contains labeled voxels that are common in Step 1 and Step 2. The number of resulting voxels is summed and multiplied by voxel dimensions to derive a total volume score. The validity and reliability of this approach has been demonstrated previously [31].

#### Cerebral blood flow

Transcranial Doppler (TCD) ultrasonography was performed through an expanded Stroke Prevention Trial in Sickle Cell Anemia (STOP) protocol [32] and used to quantify mean cerebral blood flow velocity (CBF-V) of the Middle Cerebral Artery (MCA). The MCA was chosen to operationalize cerebral blood flow in this study for several reasons. First, it irrigates the frontal, temporal, and the parietal cerebrum and reliably reflects changes in cerebral blood flow. CBF-V of the MCA also exhibits higher blood flow velocity compared to other TCD measured arteries (e.g., ACA, PCA) [33,34]. Furthermore, persons with HF demonstrate significant reductions in CBF-V of the MCA [35] and such reductions have been linked with WMH in older adults with HF [15]. Lastly, CBF-V of the MCA was also chosen because of its known sensitivity to sleep disturbances in healthy populations [36].

#### Estimate of HF severity/physical fitness

The 2-minute step test (2MST) was used to assess physical fitness levels and to serve as a proxy of HF severity in the current sample [37]. The 2MST is a brief assessment that requires participants to march in place lifting his/her knees to a marked target set on the wall set at the midpoint between the kneecap and crest of the iliac for a 2-minute period. Greater step count is associated with better physical fitness and has been correlated with metabolic equivalents derived from stress testing [38]. The 2MST was included as a covariate in the current analyses to control for the effects of physical fitness on the brain in HF [39] and on sleep quality [40].

#### Demographic and medical history

Participants’ medical and demographic history was ascertained through self-report and corroborated and supplemented by medical record review. Through these methods, information regarding participants’ physician diagnostic history of diabetes, sleep apnea, hypertension, and depression as well as prescribed medications were obtained. LVEF of the current sample was obtained through medical chart review. Medical record review took precedence in the case of discrepancy from self-report. Refer to Table 1.

### Procedures

The Kent State University and Summa Health System Institutional Review Board (IRB) approved the study procedures and all participants provided written informed consent prior to study enrollment. At a baseline assessment, all participants completed demographic, medical history, and psychosocial self-report measures, including the PSQI. A medical record review was also completed at this baseline assessment to corroborate self-report and also ascertain LVEF of the sample; all participants completed an echocardiograph as part of their standard clinical care prior to study entry. At a separate study visit, but within 2-weeks of the baseline assessment, all participants also underwent MRI and TCD ultrasonography.

### Statistical methods

All analyses were performed using SPSS software. A log transformation of WMH was performed to correct for the positively skewed distribution of this variable. A series of multivariable hierarchical regression analyses was conducted to examine the effects of adverse brain changes on sleep quality in HF. For all analyses, the global PSQI served as the continuous dependent variable. To determine the independent effects of brain indices on sleep quality, demographic and medical covariates that are known to influence neurocognitive outcomes and/or sleep quality in HF were entered into block 1 of all regression models to account for their variance. These covariates included age, LVEF, 2MST, intracranial volume, and diagnostic history of diabetes, sleep apnea, hypertension, and depression (1 = positive diagnostic history; 0 = negative diagnostic history). A separate regression model was performed for each of the following continuous brain predictors that were entered in block 2, yielding six separate regression models: WMH, total brain volume, total gray matter volume, thalamus, and brain stem volume, and CBF-V of the MCA. Intracranial volume was not controlled for in the regression model examining CBF-V of the MCA as the predictor variable, as head size does not represent a possible confound in this analysis. Follow-up partial correlations adjusting for age, LVEF, 2MST, intracranial volume, and diagnostic history of diabetes, sleep apnea, hypertension, and depression were conducted to examine the correlations among WMH, cerebral blood flow, specific item-level sleep difficulties of the PSQI.

Lastly, bivariate correlations examined the association between CBF-V of the MCA and WMH. A regression model with the above listed covariates (but, not intracranial volume) entered in block 1 and CBF-V of the MCA and WMH entered in block 2 was then performed to determine whether the combination of cerebral hypoperfusion and increased WMH independently predicted poor sleep quality.

## Results

### Sleep quality

Refer to Table 2 for sleep characteristics of the current sample. The sample had an average global PSQI score of 8.41 (SD = 3.57) with 75.5% classified as impaired sleepers (i.e., PSQI > 5). The most commonly reported sleep difficulties in the sample included interrupted sleep, nocturia, snoring or coughing fits, and pain. In terms of overall quality of sleep, 5.7% rated their quality of sleep to be very bad and 22.6% reported their sleep quality to be fairly bad.

**Table 2:** Reported sleep difficulties in 53 older adults with heart failure

	**Once or twice per week (%)**	**Three or more times per week (%)**
Cannot Get to Sleep within 30 Minutes	26.4	17.0
Wake Up in the Middle of the Night or Early Morning	30.2	41.5
Get Up to Use the Bathroom	26.4	58.5
Cannot Breathe Comfortably	7.5	11.3
Cough or Snore Loudly	7.5	26.4
Feel Too cold	1.9	13.2
Feel Too Hot	13.2	5.7
Bad Dreams	9.4	3.8
Pain	13.2	20.8
Other Reasons	11.3	9.4

### Neuroimaging, cerebral blood flow, and overall sleep quality

See Table 3 for a full summary of demographic and medical predictors of sleep quality. After adjustment for medical and demographic variables and intracranial volume, WMH was associated with the global PSQI (β = 0.28, *p* = 0.046). Increased WMH volume was associated with greater sleep difficulties. CBF-V of the MCA also emerged as a significant predictor of global PSQI (β = -0.28, *p* = 0.04), even after controlling for medical and demographic variables. See Table 4. Decreased CBF-V of the MCA was associated with poorer sleep quality. There were no significant effects on sleep quality for total brain, total gray matter volume or for any of the regional volumetric indices (i.e., thalamus or brain stem volume) (*p* > .05 for all).

**Table 3:** **WMH predict sleep quality in older adults with heart failure** (***N*** = **53**)

**Variable**	**Global PSQI**
	***β*** **(*SE b*)**
*Block 1*	
Age	-.03(.06)
2MST	-.36(.02)*
LVEF	-.10(.03)
Depression	-.06(1.25)
Hypertension	-.17(1.04)
Diabetes	.12(1.09)
Sleep Apnea	-.18(.00)
ICV	-.18(.00)
*R*^*2*^	.30
*F*	2.41*
*Block 2*	
WMH	.28(1.36)*
*R*^*2*^	.37
*F* for ΔR^2^	4.22*

*Note*. *denotes p < 0.05.

*Abbreviations*: *β* standardized regression coefficients, *SE* standard error, 2*MST* 2-minute step test, *LVEF* left ventricular ejection fraction, *ICV* Intracranial volume, *WMH* White matter hyperintensities, *PSQI* Pittsburgh sleep quality index.

**Table 4:** **Cerebral blood flow predicts sleep quality in older adults with heart failure** (***N*** = **53**)

**Variable**	**Global PSQI**
	***β*** **( *SE b *)**
*Block 1*	
Age	.06(.06)
2MST	.43(.02)*
LVEF	-.10(.03)
Depression	-.04(1.24)
Hypertension	-.15(1.03)
Diabetes	.14(1.08)
Sleep Apnea	.21(1.12)
*R*^*2*^	.28
*F*	2.53*
*Block 2*	
CBF-V MCA	-.28(.04)*
*R*^*2*^	.35
*F* for ΔR^2^	4.41*

*Note*. *denotes p <0.05.

*Abbreviations*: *β* standardized regression coefficients, *SE* standard error, 2*MST* 2-minute step test, *LVEF* left ventricular ejection fraction, *CBF*-*V MCA* cerebral blood flow velocity of the middle cerebral artery, *PSQI* Pittsburgh sleep quality index.

### Sleep difficulties, WMH, cerebral blood flow

Partial correlations adjusting for age, LVEF, 2MST, intracranial volume, and diagnostic history of diabetes, sleep apnea, hypertension, and depression showed increased WMH was associated with greater difficulty falling asleep (*r* (43) = 0.31, *p* = 0.04), feeling too hot (*r*(43) = 0.32, *p* = 0.03), and decreased overall sleep quality (*r*(43) = 0.39, *p* = .01). Lower CBF-V was also associated with having bad dreams (*r*(43) = -0.39, *p* = 0.01), decreased enthusiasm to get things done (*r*(43) = -0.40, *p* = 0.01), and poorer overall quality of sleep (*r*(43) = -0.40, *p* = 0.01). Interestingly, greater CBF-V of the MCA was associated with increased sleep medication use (*r*(43) = 0.33, *p* = 0.03).

### Association between WMH and cerebral blood flow

Bivariate correlations showed that CBF-V of the MCA demonstrated a significant association with WMH (*r*(51) = -0.31, *p* = 0.03). Decreased cerebral blood flow was associated with greater WMH. Regression analyses controlling for medical and demographic variables revealed that the combination of both reduced CBF-V of the MCA and increased WMH demonstrated significant predictive properties of poorer sleep quality (*F*(10,42) = 2.93, *p* = 0.03), suggesting a possible interaction between these variables on sleep quality.

## Discussion

Consistent with the extant literature, the current study shows that reported sleep difficulties are prevalent in older adults with HF. Past work has demonstrated that a series of demographic (e.g., older age) and medical factors (e.g., HF severity) are correlated with poor sleep quality in HF [5], though findings are not entirely consistent across studies [7]. The current findings extend past work by showing that adverse structural and functional brain changes may be important contributors to sleep disturbances in patients with HF.

The current study shows that greater WMH volume is associated with poorer sleep quality in older adults with HF. The exact mechanisms underlying this association are unclear, though there are several possible explanations. Disrupted circadian rhythms have been shown to occur in vascular dementia patients, believed to be the result of deafferentation [41,42], and WMH may lead to similar circadian rhythm disturbances in HF by interfering with cortical and subcortical neuronal connections to key brain regions that help regulate sleep (e.g., brain stem, suprachiasmatic nucleus) [43-47]. Supporting this possibility is the current association between WMH and altered core body temperature (i.e., feeling too hot as assessed by item 5 g on the PSQI) and past work that demonstrates HF patients exhibit impaired integrity of axonal projections among brain structures important for sleep, including connections among the basal forebrain, hypothalamus, raphe magnus, and brain stem [20,48]. WMH are also closely linked with amyloid beta deposition [49] and amyloid beta is a significant contributor to poor sleep quality in pre-clinical Alzheimer’s disease (AD) patients [50]. This is noteworthy, as HF patients are at risk for AD and declining sleep quality may be indicative of AD pathogenesis in this population [50,51]. Lastly, the known influence of WMH on psychiatric symptoms (e.g., depression) in HF may also contribute to decreased sleep quality [13,52].

Reduced cerebral blood flow also emerged as a significant predictor of sleep quality in the current sample of HF patients, and may interact with increased WMH to exacerbate sleep disturbances. Cerebral hypoperfusion and subsequent ischemia is commonly proposed to underlie WMH in HF [15,21] and the current findings showed an inverse association between cerebral perfusion and WMH. Thus, it is likely that chronic disruptions in cerebral hemodynamics affects sleep in HF through its association with WMH, though the cross-sectional design of the current study precludes empirical test of such mediation. Our findings are in the opposite direction of past work examining cerebral blood flow and sleep quality in AD patients [24]. Similarly, sleep spindles help to prevent arousal during sleep, and in contrast to the suggested direction of our findings, past work shows that cerebral blood flow and spindle activity are negatively correlated [25,53-56]. The exact reason for the directionality of our findings is not clear, but it is plausible that decreased cerebral blood flow in HF may reflect greater HF severity such as increased number of medical comorbidities and cardiac dysfunction, which are all significant correlates of poor sleep quality in this population [5]. Interestingly, past work shows that cerebral blood flow can be improved through physical activity interventions [57]. This is noteworthy, as improved systemic perfusion in HF has been linked with better neurocognitive outcomes [58]. Because physical activity is a key treatment recommendation in the management of HF, future studies should examine whether greater physical activity results in better sleep quality among HF patients due to improved cerebral circulation.

The current study found no association between total and regional brain volume and sleep quality. These findings further suggest interference with cortico-cortical and cortico-subcortical connections as a likely underpinning for poor sleep quality in HF given the observed impact of WMH on reduced sleep quality in the current study. Although brain atrophy has been linked with reduced sleep quality in other medical populations [19], it is likely that atrophy in the current sample did not reach threshold to produce clinical manifestations. For instance, brain volume loss has been shown among patients with greater HF severity (i.e., LVEF <30) [20] while LVEF in this sample fell within the average range (i.e., mean LVEF = 40). Longitudinal studies that examine the effects of brain atrophy on sleep in HF are needed, particularly as it involves WMH.

The current findings are limited in several ways. The cross-sectional design does not permit causal inferences and prospective studies are needed to confirm our findings. For instance, it is possible that insufficient sleep in HF may lead to reduced cerebral perfusion [23], though this is unlikely in this population in light of the negative effects of cerebral hypoperfusion on the brain [15]. In addition, self-report of sleep quality is limited by biases [59] and future work should use objective assessments of sleep quality (e.g., polysomnography). Similarly, future studies that employ imaging techniques examining axonal tractography would also help elucidate the underlying mechanisms between WMH and sleep quality through direct examination of neuronal pathways that may be occluded by the presence of WMH. We also found an inverse association between physical fitness and sleep quality. Past work shows that exercise interventions in HF lead to improved sleep quality [60] and future work should examine whether such findings are a result of the positive benefits of exercise on the brain. Lastly, although it is notable that the current study controlled for the effects of diagnosed sleep apnea, such history was obtained through self-report and medical record review and information regarding duration, treatment, and severity of sleep apnea is unknown. Consequently, it is possible that undiagnosed sleep apnea or sleep disordered breathing was present in many of the HF participants [10], which may have introduced possible confounds. Future work that employs more objective assessments of sleep quality is needed to clarify and confirm the current findings. Likewise, case controlled studies with larger samples that are more diverse in disease severity and utilize direct measurements of comorbid conditions are much needed to better understand the independent effects of brain abnormalities on sleep in HF.

## Conclusions

In brief summary, the current study shows that WMH and decreased cerebral blood flow are associated with poor sleep quality in older adults with HF. Prospective studies are needed to confirm these findings, clarify mechanisms by which WMH and reduced cerebral blood flow disrupt sleep, and examine whether sleep difficulties are modifiable through interventions that target improved cerebral blood flow (e.g., cardiac rehabilitation).

## Abbreviations

HF: Heart failure; WMH: White matter hyperintensities; LVEF: Left ventricular ejection fraction; CBF-V of the MCA: Cerebral blood flow velocity of the middle cerebral artery; 2MST: 2-minute step test.

## Competing interests

The authors declare that they have no competing interests.

## Authors’ contributions

MA, AMB, MBS, JG, RC, and LS participated in the design of the study, acquisition of data, and analysis and interpretation of data. MA, MBS, JG, EYG, AN, JH, JR participated in acquisition of data and analysis and interpretation of data. All authors were involved in drafting the manuscript and revising it for intellectual content. All authors also provided final approval of the version to be published.
